# Electrochemical Cycling of Liquid Organic Hydrogen Carriers as a Sustainable Approach for Hydrogen Storage and Transportation

**DOI:** 10.1021/acssuschemeng.4c05784

**Published:** 2025-01-15

**Authors:** Moses D. Chilunda, Sarvarjon A. Talipov, Hafiz M. Umar Farooq, Elizabeth J. Biddinger

**Affiliations:** Department of Chemical Engineering, The City College of New York, CUNY, New York, New York 10031, United States

**Keywords:** energy storage, hydrogen transport, electrochemical cycling, carbon footprint, energy efficiency, liquid organic hydrogen carriers

## Abstract

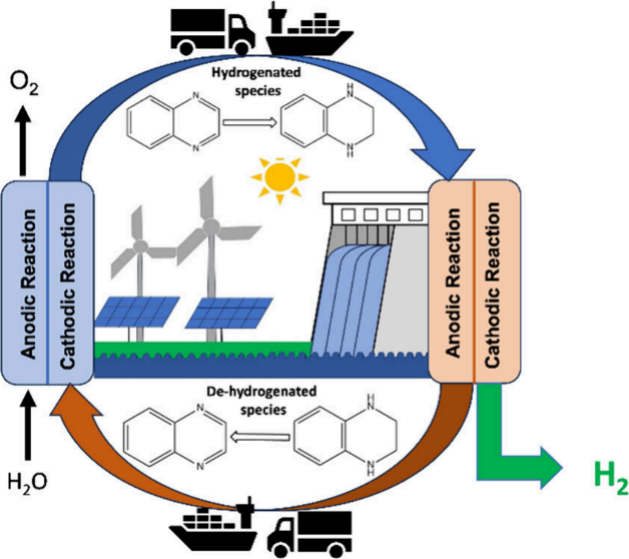

Hydrogen (H_2_), as a high-energy-density molecule, offers a clean solution to carry energy. However, the high diffusivity and low volumetric density of H_2_ pose a challenge for long-term storage and transportation. Liquid organic hydrogen carriers (LOHCs) have been suggested as a strategic way to store and transport hydrogen in stable molecules. More so, electrochemical LOHC cycling renders an opportunity to utilize renewable energy for hydrogen storage and transportation toward the goal of eliminating carbon emissions. In this Perspective, examples of electrochemical reactions of organic molecules and their suitability for LOHC couples are examined. A comparative carbon footprint assessment of electrochemical LOHC cycling processes against thermochemical and hybrid LOHC cycling processes was performed. The electrochemical LOHC cycling process had the lowest relative carbon footprint only when highly concentrated LOHCs were used as the feed or when purification of the LOHC product was not required. The carbon footprint in electrochemical cycling of diluted LOHC was primarily contributed to by the LOHC distillation separation process. A sensitivity analysis showed the carbon footprint LOHC concentration dependence during the electrochemical cycling process. Moreover, the electrolyte composition significantly affects the carbon footprint during electrochemical LOHC cycling. Energy utilization, water usage, and toxicity for electrochemical LOHC cycling are discussed to provide an overview for better economic and environmental practices. There are significant opportunities in the electrochemical cycling of LOHCs if appropriate conditions such as high concentrations of reactant, reversible redox cycling ability, high Faradaic efficiencies, and catalyst stabilities are achieved.

## Abstract 2

Electrochemical techniques provide an opportunity to develop greener methods to store and transport hydrogen via liquid organic hydrogen carriers.

## Introduction

Mitigating carbon emissions has been a great motivation for transitioning to renewable energy, resulting in a large growth of renewable energy in recent years. In 2023, 43% of the global installed power capacity was from renewable energy sources.^1^ The global renewable energy reserve in 2023 also increased by 13.9% from that in the previous year.^1^ This trend will need to continuously increase for the foreseeable future to meet the Paris agreement goals. A major concern in the growth of and reliance on renewable energy sources is the variable nature requiring storage for load leveling. Battery installations have significantly grown in recent years but are challenged when it comes to long-term storage.^2^ In the U.S., pumped hydro storage is currently the most utilized method for storing renewable energy for long-term use.^2,3^ However, pumped hydro storage faces geographical constraints to meet its specific demands for operation.^2^

Hydrogen (H_2_) has attracted attention as an alternative energy carrier. The versatility of H_2_ to be used in industry, transport, electricity generation, and storage makes it a good candidate in storing renewable energy.^4^ Compressed H_2_ gas tanks and liquified H_2_ tanks are the conventional methods for storing and transporting hydrogen.^5,6^ These conventional methods face shortcomings when it comes to long storage durations and large distance transportation because H_2_ can easily diffuse through tank walls because of its small molecular size. Moreover, liquefying hydrogen in cryogenic tanks requires a large energy input, making the process expensive. Alternative H_2_ storage methods have been investigated. Such methods include underground storage,^6,7^ metal hydrides,^5,6,8,9^ complex hydrides,^6^ metal–organic frameworks (MOFs),^8,10^ and liquid hydrogen carriers.^11−13^ Underground storage appears to be a very good way of storing hydrogen for a long period of time and has been practically applied in salt caverns.^6,7^ Nonetheless, storing hydrogen underground requires initial high capital investment, geological space nearby, and the risk of impurities to the hydrogen stored.^6,7^ Metal and complex hydrides offer an opportunity to store hydrogen with a high volumetric density; however, they have rather slow kinetics and require excessive temperatures to release hydrogen.^6^ The microporous structure of MOFs allows them to have a high surface area for hydrogen adsorption, resulting in a high hydrogen storage capacity.^10^ However, the high storage capacity of MOFs is generally possible only at very low temperatures, and novel designs are required for ambient temperature utilization.^10^ Liquid hydrogen carriers are also top candidates in storing hydrogen because they have a well-established storage and transportation infrastructure. Liquid ammonia is an example of a liquid hydrogen carrier that has a well-established transportation line and can potentially be used to store hydrogen atoms, which are bound to the nitrogen atom.^13^ Despite mature infrastructure and high hydrogen density, liquid ammonia brings up safety concerns due to its toxicity and explosive risks.^13^ Liquid organic hydrogen carriers (LOHCs) can also be used to store and transport hydrogen through a chemical reaction mechanism. With LOHCs, hydrogen is chemically bound to the organic molecular structure covalently. In this Perspective, we focus on LOHCs as a hydrogen storage and transportation method.

### LOHCs

LOHCs offer a relatively inexpensive and safe way to store and transport hydrogen.^8,11,14−27^ No formation or breaking of the original organic chemical backbone during the cycling occurs with LOHCs. The LOHC process method is based on the catalytic hydrogenation of hydrogen-lean organic molecules, where hydrogen is stored in a formed hydrogen-rich molecule. The hydrogen-rich molecule can be stored in existing storage infrastructures or transported to a desired location for hydrogen release. The hydrogen release process occurs via a catalytic dehydrogenation process of the hydrogen-rich molecule. For notation convenience, LOHC couples in this work will be referred to in the form of hydrogen-lean/hydrogen-rich pairs. An important aspect of LOHCs is the functional group undergoing a hydrogenation/dehydrogenation process. The number of hydrogen atoms transferred during hydrogenation/dehydrogenation reactions will depend on the functional groups undergoing the chemical reaction. The amount of hydrogen transferred, together with the molecular weight of the LOHC, will also determine the hydrogen storage capacity of an LOHC molecule. Table 1 presents LOHC couples discussed in this Perspective, along with their hydrogen storage capacity, standard Gibbs free energy (Δ*G*°), and standard redox potential (*E*°). For a comparison of the LOHC couples, the Δ*G*° per mole of H_2_ transferred (Δ*G*° relative) is also included.

**Table 1:**
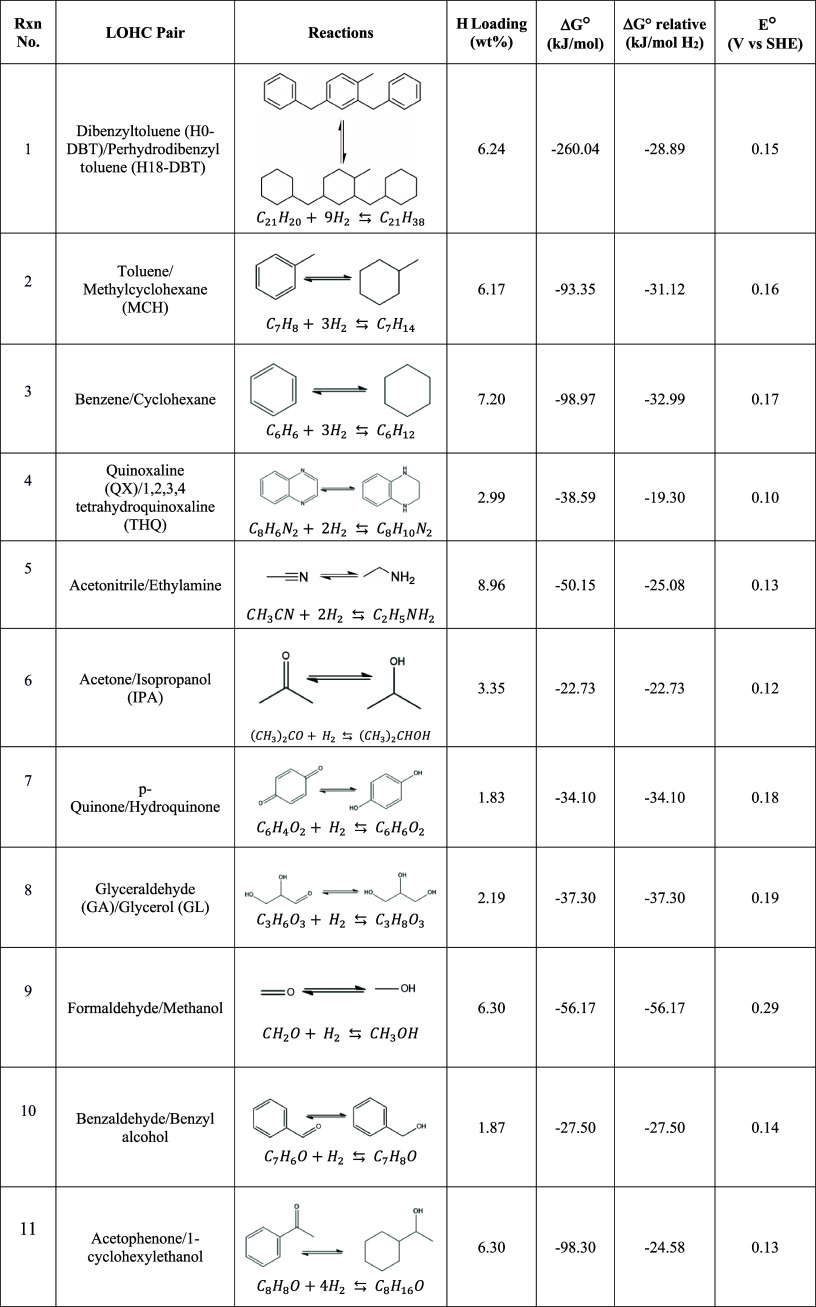
LOHC Couples and Some of Their Properties

An example of an LOHC couple is the dibenzyltoluene/perhydrodibenzyltoluene pair (H0-DBT/H18-DBT).^17,18^ The H0-DBT/H18-DBT couple offers a hydrogen storage capacity of 6.24 wt %, making it an efficient system for reversible hydrogenation and dehydrogenation cycles (reaction 1).^23^ One key advantage of the H0-DBT/H18-DBT couple is the wide liquid range of the pair molecules, with melting points (MPs) below −30 °C and boiling points (BPs) greater than 350 °C, ensuring stability and ease of handling in various operating conditions.^11,28^ One notable drawback of the DBT-based system is its high energy consumption during the thermochemical dehydrogenation (TCD) process, which can impact the overall efficiency of the hydrogen storage and release.^29^ Additionally, the need for purification of hydrogen gas released during dehydrogenation due to potential impurities or byproducts generated in the process adds complexity and cost to the overall operation.^17,29^

A notable demonstration of LOHC was performed by Chiyoda Corp. and involved the toluene/methylcyclohexane (TOL/MCH) LOHC pair (reaction 2) for thermocatalytic cycling.^30,31^ The TOL/MCH system exhibited a robust performance throughout the demonstration period. The pilot plant in Japan had a capacity of 50 N·m_H_2__^3^/h and operated from April 2013 to November 2014.^30,31^ Operating at pressures lower than 1 MPa, the hydrogenation process maintained toluene conversion rates above 99% and MCH selectivity above 99%.^30^ The dehydrogenation process achieved MCH conversion rates above 95% and TOL selectivity above 99%, with hydrogen and toluene yields surpassing 95%.^30^ In 2020, Chiyoda Corp. further demonstrated long-distance hydrogen transportation from Brunei to Japan using the LOHC concept.^32^ These results underscored the efficiency and effectiveness of the LOHC system as a hydrogen storage and transport technique at a demonstration scale.

In general, any unsaturated organic molecules that can reversibly undergo hydrogenation reactions can be considered as an LOHC molecule. However, some LOHC couple candidates are theoretically more promising compared to others, accounting for factors such as thermodynamic properties and hydrogen storage capacity.

### Methods of LOHC Cycling

**Figure 1:**
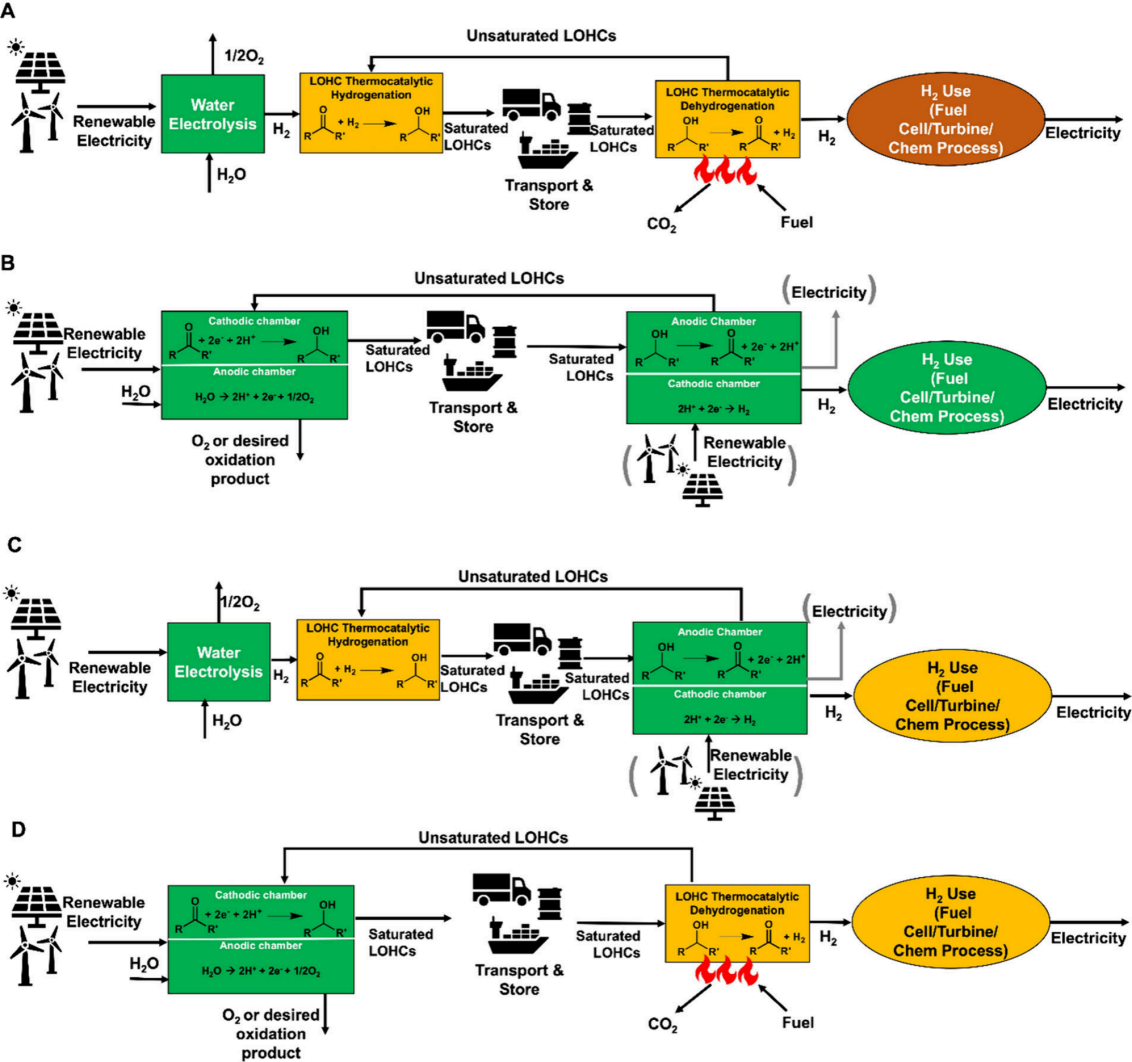
LOHC cycling process for (A) thermochemical, (B) electrochemical, (C) hybrid scenario 1, and (D) hybrid scenario 2. Parentheses are included around the ECD renewable energy and electricity components to indicate that the ECD reactor may require or generate electricity depending on the redox potential and the kinetics (overpotential) of the couple used.

Thermochemical hydrogenation and dehydrogenation of the LOHC molecules are the primary methods of cycling proposed in the literature for the utilization of LOHCs for hydrogen storage and transportation.^11,14−27,29^ In the thermochemical cycling, generally, water electrolysis (or methane steam reforming with CO_2_ capture) will feed hydrogen to a thermochemical hydrogenation (TCH) reactor (Figure 1A). TCH is an exothermic reaction that often needs cooling to maintain the desired reaction temperatures for maximum product selectivity and longer catalyst life. Higher hydrogen feed pressure is also desired to drive the reaction equilibrium forward for the TCH reactions. When hydrogen is needed, the hydrogenated LOHC molecule releases hydrogen during the TCD reaction. The dehydrogenation reaction is endothermic and requires high-temperature conditions to occur. Also, the TCD reaction usually operates at low-pressure conditions to catalytically dehydrogenate the hydrogen-rich molecule back to its original hydrogen-lean form.^27^ Significant concerns associated with thermochemical cycling of LOHCs include the high operating and energy costs for the thermochemical reactions occurring at elevated temperatures. High energy costs also lead to CO_2_ emissions contributed during the dehydrogenation reactions from conventionally heating the reactors. While thermochemical cycling offers a pathway for renewable hydrogen integration, its reliance on high dehydrogenation temperatures underscores the importance of mitigating CO_2_ emissions and minimizing the environmental impact in the pursuit of sustainable energy solutions. Moreover, thermochemical reactors require extended periods to heat up and cool down, hindering their dynamic performance.

In addition to the thermochemical cycling of LOHCs, electrochemical cycling of LOHCs has been gaining attention in the literature.^33−41^ In electrochemical LOHC cycling, the hydrogen-lean molecule is hydrogenated via electrochemical hydrogenation (ECH) reactions on the cathode to form a hydrogen-rich LOHC molecule. Simultaneously, the oxygen evolution reaction (OER) or any other desired oxidation reaction takes place on the anode. Molecular hydrogen is not needed for ECH because protons can be sourced in situ from the anode reaction during LOHC ECH, thereby eliminating a unit operation in the LOHC cycle. The electrochemical dehydrogenation (ECD) reaction of the hydrogen-rich molecule occurs on the anode, while simultaneously hydrogen evolution reaction (HER) occurs on the cathode releasing hydrogen (Figure 1B). Depending on the redox potential and kinetics (overpotential), the ECD reactor can operate electrolytically (consuming electricity) or galvanically (generating electricity as a fuel cell), as shown in Figure 1B,C. The application of direct LOHC fuel cells has been reported in the literature.^33,39,42^ The primary advantage of a direct LOHC fuel cell is that it eliminates the need to handle molecular hydrogen for electricity generation, thereby simplifying the process complexity, operating costs, and safety risks.

Hybrid cycling, an innovative approach in the realm of LOHCs, combines thermochemical and electrochemical processes to enhance hydrogen storage and release capabilities. Hybrid cycling leverages the advantages of both electrochemical and thermochemical techniques to overcome reaction limitations and improve the system performance. In the first hybrid LOHC cycling scenario (Figure 1C), an endothermic TCD reaction is replaced by an ECD reaction happening at ambient conditions. The ECH reaction can replace the TCH reaction, which may require higher temperatures and pressures (Figure 1D). For strategic energy transport, electrochemical reactions can be conducted at locations with abundant renewable energy for utilization. Additionally, Sievi et al. proposed a hybrid LOHC cycling system integrating a H-18 DBT TCD process with an isopropyl alcohol (IPA) direct alcohol fuel cell (DAFC).^33^ In their study, acetone produced from IPA ECD in the DAFC was thermochemically hydrogenated back to IPA where hydrogen was sourced from dehydrogenation of H-18 DBT.^33^ The novel hybrid cycling system was observed to remarkably decrease the dehydrogenation temperature conditions in the TCD of H-18 DBT.^33^ Hybrid cycling of LOHCs has promise for advancing the efficiency, kinetics, and sustainability of LOHC-based hydrogen storage and transportation systems. While hybrid cycling of LOHCs holds promise for enhancing hydrogen storage and release capabilities, its feasibility is currently limited by technical challenges, few research studies, and complexity compared with thermochemical and electrochemical cycling methods.

The significant benefits offered by the electrochemical approach over thermochemical LOHC and the early-stage technology for electrochemical LOHC cycling provide a need to improve electrochemical LOHC cycling technology. The subsequent focus in this work will be on highlighting the key areas for electrochemical LOHC cycling to promote further research and development. This work analyzes electrochemical reactions of prospective LOHC candidate molecules, determining the strengths and areas of improvement for optimal reaction performance in LOHC cycling applications. Furthermore, key areas for improving greener LOHC cycling processes are evaluated through a comparative carbon footprint analysis of LOHC cycling systems, along with an overview of energy efficiency, LOHC molecule toxicity, and water usage in electrochemical LOHC cycling systems.

## LOHC Candidate Groups for Electrochemical Cycling

Electrochemically activatable organic molecules that can undergo redox reactions to store and release hydrogen are required to pave the way for the development and viability of electrochemical LOHC cycling applications.^34,40^ This section discusses the ECH and ECD reactions of LOHC pairs for electrochemical cycling, categorizing candidate molecules by functional groups that undergo these reactions. The electrochemical reactions will be discussed from fundamental to practical perspectives and insights into developing electrochemical cycling systems.

### Criteria for LOHC Candidates

A key criterion for a good LOHC candidate for electrochemical cycling applications is its structural reversibility under electrochemical redox reactions to ensure efficient hydrogen utilization and storage. Additionally, the candidate pair should exhibit a high hydrogen density to enhance the storage and utilization efficiency. Similar to the thermochemical process for LOHC cycling, the compatibility of the candidate with well-designed electrocatalysts is crucial to achieving high conversions, product selectivity, and faradaic efficiency (FE). A good LOHC candidate should also exhibit minimal side reactions during electrochemical processes to ensure high efficiency and reusability. Challenges such as electrode poisoning and polymerization side reactions hinder the effective utilization of electrochemical cycling of LOHC. In addition, energy losses associated with electrochemical reactions due to high overpotentials and ohmic drops hinder electrochemical cycling of LOHCs from wide implementation in chemical industry. Further studies are required to improve the technological immature electrochemical reactions from the fundamental to the practical level. Thus, understanding the kinetics and mechanisms of ECH and ECD reactions together with developing effective catalytic systems are crucial for industrial application of electrochemical LOHC cycling.

### LOHC Candidates According to Functional Groups

The functional group that is hydrogenated/dehydrogenated in the organic molecule influences the hydrogen loading capacity, kinetics, stability, types of catalysts, and many more properties. Herein, promising LOHC candidates are discussed by grouping them according to their functional groups that undergo the hydrogenation/dehydrogenation cycle. The emphasis on the LOHC candidates reviewed spans fundamental to practical aspects from reaction mechanisms to reaction performance to better understand and develop efficient ways of electrochemical cycling.

### Aromatic Ring Saturation

Ring saturation is a promising approach for hydrogen storage involving the addition of hydrogen atoms to unsaturated cyclic compounds to form saturated ring structures. Quinoxaline (QX) is a bicyclic aromatic compound that offers a safer alternative to hydrogen storage. Ring hydrogenation of QX involves the addition of four hydrogen atoms (Reaction 4) to form 1,2,3,4-tetrahydroquinoxaline (THQ), which can be further electrochemically dehydrogenated to release four hydrogen atoms.^43^ QX is attractive for hydrogen storage and transport applications due to its cyclic structure, which provides stability.^44−46^

#### Quinoxaline/1,2,3,4-Tetrahydroquinoxaline (QX/THQ)

**Figure 2:**
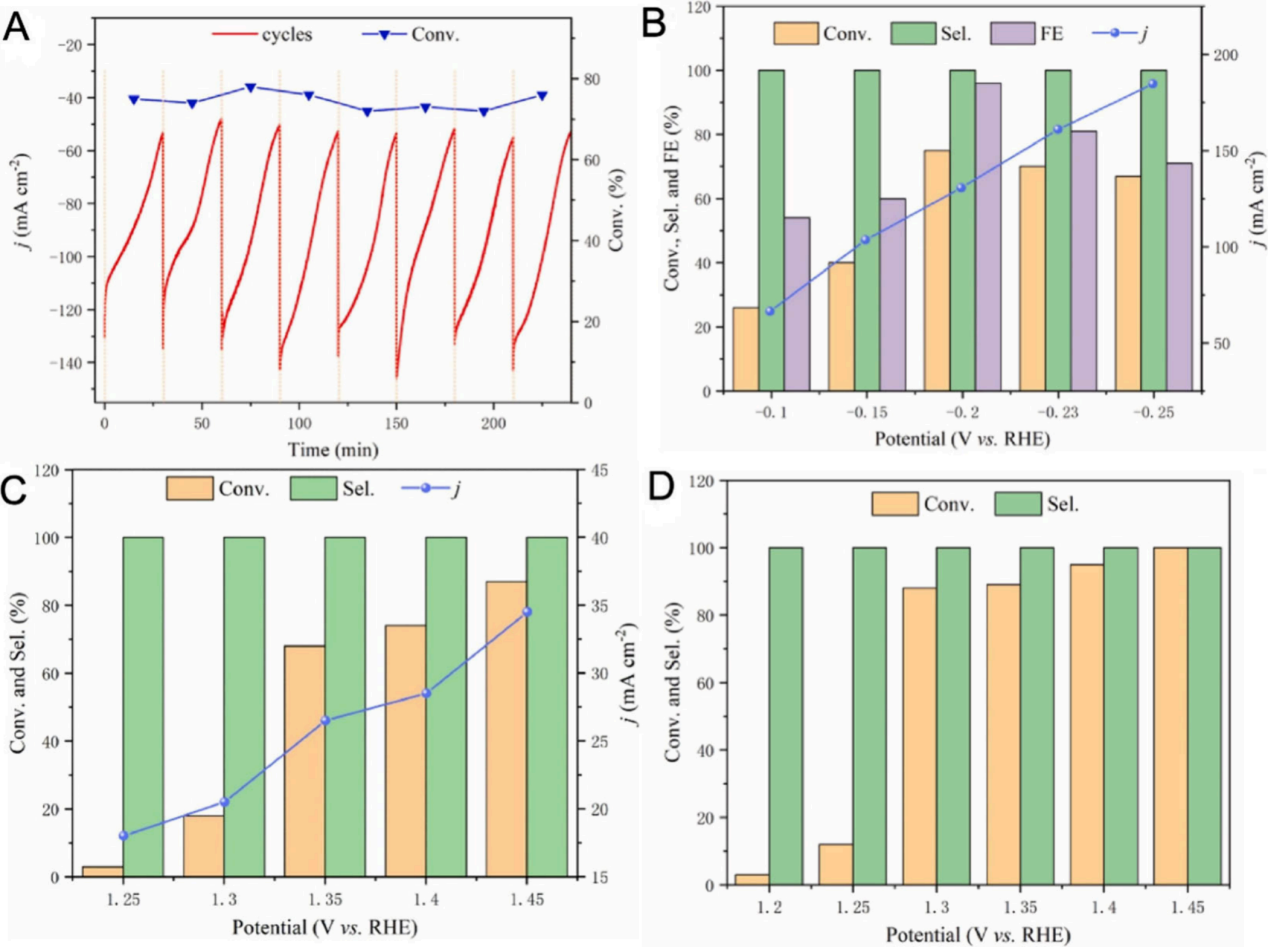
(A) ECH of 12.5 mM QX on Pd/NF as a function of the reaction time under −0.2 V vs RHE and 1 M KOH solution, performed in 8 cycles.^45^ (B) Conversion, selectivity, and current density of 12.5 mM QX ECH at different cathodic potentials on Pd/NF catalyst and 1 M KOH solution. The reaction time is 30 min. Reprinted with permission from ref (45). Copyright 2024 Elsevier. (C) Conversion, selectivity, and current density of 5 mM THQ ECD at different anodic potentials on P-doped WO_3_/NF and 1 M KOH solution. The reaction time is 15 min. Reprinted with permission from ref (44). Copyright 2024 Elsevier. (D) Conversion and selectivity of 5 mM THQ ECD at different anodic potentials on Pd/NF and 1 M KOH solution. The reaction time is 20 min. Reprinted with permission from ref (45). Copyright 2024 Elsevier.

QX ECH has been reported to produce THQ with 100% selectivity and FE over 80%.^44−46^ The ability to selectively produce THQ is promising for LOHC cycling due to product stability and the lack of side products that can affect cycling duration. Wang et al. have investigated dual-function electrocatalysts that enable ECH and ECD of the QX/THQ couple using the same catalyst.^44,45^ This would be advantageous in LOHC cycling processes because the same reactor could possibly be used for hydrogen storage and release. Moreover, the electrocatalysts have shown promising stability after 8 cycle runs, as shown in Figure 2A. Under alkaline conditions, increasing the applied cathodic potential from −0.1 to −0.25 V vs reversible hydrogen electrode (RHE) did not significantly affect the THQ selectivity, but optimal conversion and FE were observed at −0.2 V vs RHE (Figure 2B).^45^ Thus, the applied potential will be a key parameter to optimize QX ECH for the LOHC cycling process. However, QX ECH was performed under low QX concentrations (12.5 mM), leading to parasitic HER at longer reaction durations.^45^ Also, low concentrations reduce the hydrogen storage capacity of LOHC molecules.

Wang et al.’s ECD of THQ investigations showed that QX was obtained as the only product.^44,45^ Similar to ECH of QX, varying the applied potential in THQ ECD did not result in significant changes in the product selectivity under alkaline conditions.^44,45^ Nevertheless, the conversion rates increased when a positive anodic potential was applied in both P-doped WO_3_/nanofiber (NF)^44^ and Pd/NF^45^ catalysts, as shown in parts C and D of Figure 2, respectively. A significant increase in conversion was observed at 1.35 V vs RHE on P-doped WO_3_/NF,^44^ while it was similarly observed at 1.3 V vs RHE on Pd/NF (Figure 2D).^45^ Applying further positive potential gradually increased the conversion up to values greater than 90%.^44,45^ Thus, the reaction performance can be improved by further applying a positive potential in THQ ECD. However, the OER can occur as a side reaction at high anodic potentials, reducing the efficiency of the reaction.

In general, the QX/THQ couple is a promising candidate for electrochemical LOHC cycling due to its excellent reversibility, minimum side reactions, and molecular stability. Importantly, ECH and ECD of the QX/THQ couple have been demonstrated to occur using the same electrocatalysts and electrolyte composition, making the couple suitable for LOHC cycling. Further studies to understand the QX/THQ reactions at higher concentrations are needed for improvement in LOHC cycling applications in energy transportation.

Another important aromatic ring saturation LOHC couple for electrochemical cycling is the TOL/MCH couple (reaction 2). The couple has been widely studied for the thermochemical LOHC cycling, and there are limited studies in electrochemical systems. Nevertheless, studies have reported the ECH of TOL at room temperature using surfactants in aqueous electrolyte^47^ and using neat TOL.^48^ The catalyst structure affects the product distribution during the TOL ECH. MCH selectivity and FE is improved by increasing the surface roughness, while further hydrogenolysis of MCH is better on smooth Pt surfaces, forming cyclohexane and CH_4_ side products.^47^ Stankovic et al. were able to perform neat TOL ECH for 24 h using a Pd membrane electrochemical reactor.^48^ In their study, TOL ECH was performed at room temperature with the support of Rh/C on a Pd cathode to facilitate aromatic TOL adsoprtion.^48^ They did not observe any organic crossover in the membrane using nuclear magnetic resonance (NMR) spectroscopy to analyze electrolyte samples.^48^ The Pd membrane reactor creates an opportunity to overcome challenges such as organic crossovers, which typically affect the energy efficiency of electrochemical systems. The MCH ECD still requires more research due to kinetic challenges and reaction complexity, and there is room for innovative methods to improve the reaction. Benzene is another example of a LOHC molecule that can undergo ECH to form cyclohexane (reaction 3).^49,50^ However, benzene’s toxicity creates a safety concern, making it unattractive for LOHC cycling applications.^51^

### Nitriles/Primary Amines

Molecules containing a nitrile functional group can store four hydrogen atoms via ECH to form primary amines and release hydrogen via ECD of the primary amine to form the nitrile group molecule. Taking acetonitrile/ethylamine (reaction 5) as an example, the ECH and ECD reactions of nitriles and primary amines, respectively, are discussed to provide an overview and perspective of the nitrile/primary amine LOHC couples.

#### Acetonitrile/Ethylamine

One of the significant advantages of an acetonitrile/ethylamine LOHC couple is the high hydrogen storage capacity of 8.96 wt %. Furthermore, the low standard potential facilitates its thermodynamic reversibility, making it suitable for LOHC cycling applications.

**Figure 3:**
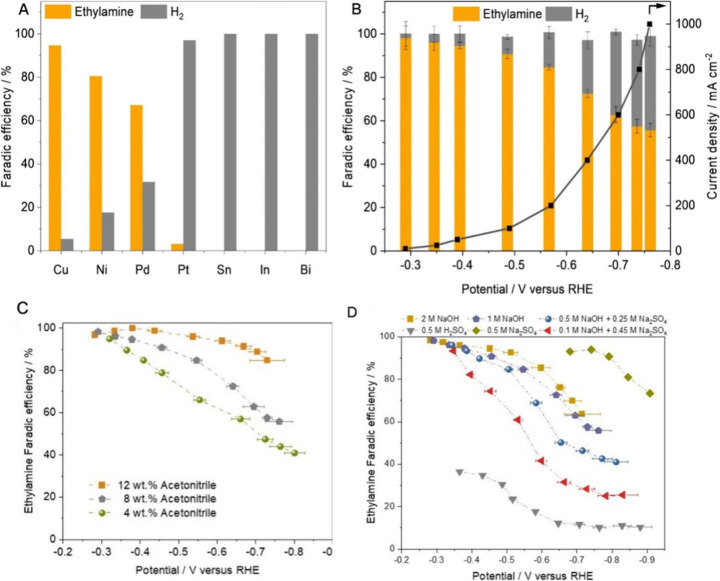
(A) Optimal FEs of ethylamine for different metal catalysts at various applied potentials during acetonitrile ECH with 1 M NaOH electrolyte.^52^ (B) Effect of the applied potential on the current density and product distribution for 8 wt % acetonitrile ECH in 1 M NaOH on Cu nanoparticles.^52^ (C) Effects of the acetonitrile initial concentration on ethylamine FEs during acetonitrile ECH at different potentials in 1 M NaOH.^52^ (D) Role of the electrolyte pH on the FEs of ethylamine at different potentials in 8 wt % acetonitrile ECH. Reprinted with permission from ref (52). Copyright 2021 Springer Nature.

ECH of acetonitrile has been reported to form ethylamine as the main product, with selectivity values greater than 90% favoring its LOHC application.^38,52−55^ High ethylamine selectivity offers acetonitrile ECH an advantage over the respective TCH process, forming significant amounts of dimers and trimers (about 10–40% selectivity) as major byproducts.^52,55−57^ Under alkaline conditions, Cu catalysts have had the best results for acetonitrile ECH compared to other metal catalysts.^38,52^Figure 3A provides a comparison of metal catalysts that can achieve the highest ethylamine FE during acetonitrile ECH at various potentials. Tang et al. observed Pd to exhibit the highest ethylamine FE of around 60% in acidic electrolyte, with dimerization products having FE less than 8% and the rest of the electrons being consumed by HER.^55^ Under alkaline conditions, increasing the potential has been reported to increase the current density for acetonitrile ECH but at the same time decrease the FE for ethylamine, as seen in Figure 3B. In neutral conditions, increasing the potential from −0.3 to −0.5 V vs RHE increased the ethylamine FE from 88 to 94%.^53^ However, a further increase of the potential to −1.1 V vs RHE showed a decline in the FE to 79%.^53^ Similarly, in acidic conditions, increasing the potential from −0.15 to −0.75 V raised the ECH current density and ethylamine FE, with HER dominating at higher cathodic potentials.^55^ Therefore, proper tuning of the applied potential will enable the achievement of high acetonitrile ECH performance for LOHC cycling. Increasing the acetonitrile concentrations from 4 to 8 wt % in 1 M NaOH favored higher current density and ethylamine FE, as shown in Figure 3C. Higher acetonitrile concentrations and current densities are favored for LOHC cycling application, increasing the hydrogen storage capacity and reaction rates. Moreover, studies have observed lower current densities in polarization curves when the acetonitrile concentration was lowered from 2 to 0.1 M in a 1 M NaOH electrolyte.^38^ However, in 0.5 M KHCO_3_, the ethylamine FE decreased by 30% when the acetonitrile concentration increased from 1 to 5 M.^53^ The electrolyte thus also plays a major role in acetonitrile ECH, and Figure 3D provides a comparison of different electrolytes in acetonitrile ECH. It was observed that increasing the electrolyte pH favors higher current densities^38^ and ethylamine FE^52^ during acetonitrile ECH.

Ethylamine ECD has also been studied and could be used for LOHC cycling applications. Acetonitrile has been obtained as the main product for ethylamine ECD on Pt,^37,38^ making the reaction suitable for LOHC cycling. Proper tuning of the applied potential in the ethylamine ECD can offer an opportunity to improve the reaction for LOHC cycling applications. In 1 M KOH with a Pt catalyst, increasing the applied cell potential from 0.1 to 0.6 V was seen to favor higher acetonitrile and H_2_ production rates.^37^ Nevertheless, the high applied potential can reduce the product selectivity because an acetamide side product was observed at 0.8 V.^37^ Ethylamine ECD can also be optimized for LOHC cycling applications by the initial concentration of the reacting substrate and the electrolyte pH and composition.^37,38^

Overall, the acetonitrile/ethylamine couple could serve as a good electrochemical LOHC cycling couple due to the high hydrogen storage capacity, high product selectivity and FE, and low applied potentials. On the other hand, ethylamine has a lower BP (16.6 °C) and exists as a gas under room temperature, complicating its handling and cycling efficiency. Acetonitrile/ethylamine can therefore be used as a good study model for molecules with similar structures rather than as a viable couple for implementation. Another concern for this couple is the safety of acetonitrile, which has a low BP and can produce toxic volatile organic compounds (VOCs).^58^ Further investigation on organic molecules with similar structure will significantly help the LOHC field grow toward sustainable hydrogen storage. ECD of other primary amines such as 1,6-hexanediamine has been reported for LOHC cycling applications with adiponitrile obtained as the main product.^59^

### Ketones/Secondary Alcohols

A ketone functional group can store two hydrogen atoms via ECH to form a secondary alcohol group, which can release the two hydrogen atoms via ECD. The low number of hydrogen atoms transferred contributes to the relatively low hydrogen storage capacity of ketone/secondary alcohol couples. Despite the lower storage capacity, factors such as low toxicity, high product selectivity, and molecular stability offer a promising application for these couples in electrochemical cycling of LOHCs. Moreover, the ketone/secondary alcohol can be applied in stationary storage, where the reactor/tank size is not the limiting factor. Therefore, this section discusses the ECH and ECD of ketones and secondary alcohols, respectively, to provide the benefits and limitations required to improve the reactions of these couples. The acetone/IPA couple is used as the model system due to well-studied electrochemical reactions.

#### Acetone/IPA

**Figure 4:**
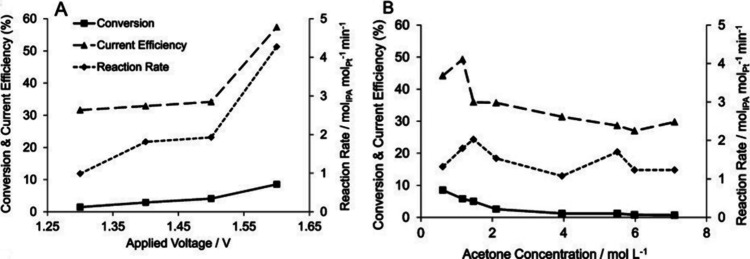
(A) Effect of the applied potential on 2 M acetone ECH in a PEM flow reactor.^64^ (B) Acetone ECH performance as a function of the acetone concentration at 1.5 V in a PEM flow reactor.^64^ Pt catalyst and 30 min residence time. Reprinted with permission from ref (64). Copyright 2012 Wiley-VCH Verlag GmbH & Co. KGaA.

The electrochemical redox couple of acetone/IPA (reaction 6) has been suggested to be a good couple for electrochemical cycling of LOHC due to its good molecular stability and low standard redox potential.^34^ Acetone ECH has been reported to form IPA under both acidic and alkaline conditions.^60−63^ For the LOHC cycling applications, the presence of side products should be avoided to obtain a good hydrogen loading efficiency. Green et al. performed a kinetic study on the ECH of acetone in a proton-exchange membrane (PEM) reactor.^64^ In their work, IPA was obtained with 100% selectivity on the Pt–Ru catalyst.^64^ They also observed an improvement in the conversion and reaction rates when the applied cell potential was increased, as shown in Figure 4A. The structure of the catalyst also affects the product formation and distribution in acetone ECH. Propane or IPA could be formed depending on the different structural orientations of Pt single crystals.^61^ For an electrochemical cycling process, the LOHC is reacted in an electrolyte solution, lowering the hydrogen storage capacity of the cycling system. For example, the use of water decreases the hydrogen storage capacity, going from 3.3 wt % for pure acetone to 0.2 wt % for aqueous 1 M acetone. Increasing the concentration of acetone from 0.5 to 6 M has shown to decrease the conversion and current efficiency of the reaction (Figure 4B).^64^ This is due to a possible blockage of active sites for proton adsorption in the catalyst surface from the suggested reaction mechanisms.^63−65^ It should also be noted that the presence of HER on the cathode as a side reaction contributes to the low current efficiency of acetone ECH.^64^ Operating at high concentrations and low HER overpotentials will likely aid in improving the reaction efficiency. Additionally, the catalyst material is a factor determining the acetone ECH performance. Benziger et al. reported a 10 times lower reaction rate of acetone ECH on Cu compared to that on Pt.^66^ There is a need to investigate cheaper and more sustainable catalysts for this reaction to be viable for an LOHC system.

The IPA ECD reaction has been studied extensively, and a high selectivity to acetone^35,42,67−72^ has been reported, favoring the electrochemical LOHC cycling process. IPA electrooxidation has largely been studied for applications in DAFC,^70,73,74^ where CO_2_ is the desired product. However, recent studies have highlighted the possible use of IPA ECD for LOHC cycling due to its high stability against C–C bond scission.^33,35,41,42,67,75,76^ The type of catalyst plays an essential role in the efficient performance of IPA ECD for hydrogen release in LOHC cycling. Bimetallic Pt–Ru exhibited greater activity and stability than monometallic Pt catalysts.^67,68^ Pd was observed to resist poisoning better than Pt catalyst in alkaline conditions.^69^ Au also had better activity in neutral and alkaline electrolytes compared to an acidic electrolyte.^69,77^ Moreover, IPA ECD studies have shown promising reactivity, where a maximum current density of ∼200 mA/cm^2^ could be obtained on Pt–Ru catalyst and acidic conditions.^67^ Despite the promising reaction kinetics for IPA ECD on Pt-based catalysts, catalyst deactivation has widely been reported as a challenge.^42,67,69,70^ Recently, Simanenko et al. identified Pt nanoclusters stabilized by Ru as the active sites for IPA ECD rather than the PtRu ensemble reported by earlier works.^75^ Understanding the mechanistic and electronic features on the catalyst interface will help to improve the IPA ECD by improving the catalyst activity and lifetime. IPA ECD studies on nickel foam did not show a significant change of the current during a constant potential electrolysis for 600 min in an alkaline medium.^72^ Generally, the catalyst stability for IPA ECD is a key piece to ensuring maximum conversion and selectivity for effective hydrogen release in an LOHC cycle system. The role of the electrolyte pH on IPA ECD will determine optimal conditions for LOHC cycling applications. Alkaline conditions offer a wide range of catalyst choice^35^ and low overpotentials^70^ compared to acidic conditions. Furthermore, studies revealed the increase of the peak current density obtained from cyclic voltammetry (CV) from 2 to 3 orders of magnitude as the pH increased from 1 to 13.^77^ Nevertheless, it is worth stating that the highest performances have been reported on acidic rather than alkaline conditions.^35^

#### Other Ketone/Secondary Alcohol Couples

Apart from the acetone/IPA couple, other ketone/secondary alcohol couples have been studied and can be used as LOHCs for electrochemical cycling. Quinones are a special group of ketones with a carbonyl as part of an aromatic ring. ECH of *p*-quinone has been demonstrated to reversibly form a corresponding secondary alcohol (hydroquinone),^78,79^ as shown in reaction 7. More studies are needed to improve the electrochemical reaction performances of the *p*-quinone/hydroquinone couple for electrochemical cycling. Overall, ketones/secondary alcohol couples show promising applications for electrochemical LOHC cycling due to their good reversibility, environmental friendliness, and good stability. The major shortcoming associated with these couples is the low hydrogen storage capacity compared to those of other LOHCs. Further analysis of organic molecules that can undergo electrochemical redox reactions on their ketone and alcohol functional groups will be required.

### Aldehydes/Primary Alcohols

The aldehyde/primary alcohol couple involves the transfer of two hydrogen atoms during electrochemical LOHC cycling. ECH of aldehydes has been extensively studied for biomass valorization,^80,81^ and the applications could be extended for LOHC usages. In this section, we cover the application of molecules with an aldehyde/primary alcohol redox structure for electrochemical cycling of LOHC, taking glyceraldehyde/glycerol (GA/GL; reaction 8) as a model for the system.

#### GA/GL Couple

GL’s nontoxic nature and biodegradability makes it an environmentally friendly substance. GA ECH can occur at alkaline conditions, with GL reported as one of the products formed.^82−84^ Under alkaline conditions, the increase of GL formation could also be observed with increasing negative potential. The effect of applied potential exerted more effect on the pH 9.7 electrolyte buffer than the pH 11.4 electrolyte buffer.^82^ Few studies have been conducted for GA ECH, and there is a need for more studies to further understand and develop the reaction for LOHC cycling applications.

GL is one of the most electrochemically active compounds among C3-saturated alcohols.^85^ GL electrooxidation can produce two different products: dihydroxyacetone and GA.^82,86−88^ There have been multiple reports on the significance of the electrocatalyst structure in the GL electrooxidation, whereby the catalyst structure can be tuned to significantly increase selectivity toward GA.^89,90^ Increasing the applied potential was observed to negatively affect GA selectivity on Pt-based catalysts.^87,91^ Tuning the cell potential will be critical in GL ECD for the LOHC cycling process. The GL initial concentration also showed an impact on GA selectivity. Zhou et al. reported that Pt/C catalyst achieved 92.6% GA selectivity using 0.05 M GL (pH 11, 0.5 M KOH, 0.7 V vs RHE), but selectivity dropped to 20% using 2 M GL.^92^ Operating at low concentrations acts as a major shortcoming for GL ECD considering it already has a low hydrogen storage capacity (2.19 wt %). Low concentrations will further reduce the capacity to release hydrogen during the cycling process. Furthermore, Kim et al. reported GA selectivity of 48.8% with 0.1 M GL at pH 0 and 0.697 V vs SHE on Pt/C.^87,88^ This implies that conversion and selectivity toward GA is higher in more alkaline conditions.

Generally, GA/GL is a promising LOHC couple for electrochemical cycling applications because the molecules are environmentally friendly. The major shortcomings of GA/GL include the low hydrogen storage capacity, high MP for GA, and the formation of side products. Due to the low hydrogen storage capacity, the GA/GL couple can be utilized specifically for stationary storage use. GA ECH is currently not well understood due to few literature reports. GL was only observed under alkaline conditions with poor selectivity due to the formation of other products obtained from dihydroxylation (C–OH cleavage) of the alcohol group in GA. GL ECD has been studied, but further developments to achieve high GA selectivity and FE at higher concentrations and conversion rates are needed.

#### Other Aldehyde/Primary Alcohol Couples

Other aldehyde/primary alcohol group molecules have been studied for electrochemical reactions and appear to be good candidates for electrochemical LOHC cycling. Formaldehyde/methanol (reaction 9) has a high hydrogen storage capacity (6.2 wt %), and the ECH of formaldehyde has been reported to achieve 96% FE to methanol on Pt single atom catalysts.^93^ ECD of methanol has also been reported with over 90% formaldehyde FE on Pd.^94^ Although formaldehyde/methanol offers high hydrogen loading capacity and electrochemical activity, environmental concerns of this couple arise due to the toxic effects and volatility of formaldehyde.^95,96^ A benzaldehyde/benzyl alcohol couple (reaction 10) has also shown good electrochemical redox ability. Benzaldehyde ECH has been extensively studied for biomass valorization, forming benzyl alcohol as a main product on various metal catalysts.^97−102^ Benzyl alcohol electrooxidation has also been reported under alkaline conditions.^103−109^ Benzaldehyde is formed as an intermediate product during benzyl alcohol electrooxidation. The electrolyte condition, catalyst, and applied potential have all shown to enhance the selectivity of benzaldehyde, allowing for benzyl alcohol ECD for LOHC cycling applications. The benzaldehyde/benzyl alcohol pair has a low hydrogen storage capacity (1.87 wt %), which is a key limitation, but serves as a useful model for studying aldehyde/primary alcohol couples.

In general, the formation of side products due to carbon bond cleavage^80,82,110^ or dimerization^80,111,112^ during the ECH of aldehydes can hinder the overall efficiency of LOHC cycling for aldehyde/primary alcohol pairs. Similarly, in the ECD of primary alcohols, the tendency of aldehydes to undergo further oxidation under certain conditions poses a challenge for maintaining the LOHC cycling efficiency. Furthermore, the studied aldehyde/primary alcohol couples have shown low hydrogen loading capacity with the exception of formaldehyde/methanol. This particular pair exhibits high storage capacity and excellent product selectivity, but formaldehyde’s environmental and health concerns make it a less favorable option. Exploring molecules with multiple electrochemically active carbonyl groups may offer a promising route toward practical electrochemical LOHC systems.

### Multiple Functional Groups

Most of the electrochemically active LOHC couples have low hydrogen storage capacity due to the low number of hydrogen atoms transferred in their functional groups. Further exploration of molecules with more than one functional group could improve the hydrogen storage capacity of LOHCs for electrochemical cycling applications. In this section, the acetophenone/1-cyclohexylethanol couple (reaction 11) is discussed as an example of an LOHC couple that can undergo electrochemical redox reaction at two functional groups.

#### Acetophenone/1-Cyclohexylethanol

Recently, Fusek et al. reported on the utilization of 1-cyclohexylethanol for direct LOHC fuel cell application.^39^ 1-Cyclohexylethanol was demonstrated to undergo ECD on its secondary alcohol and cyclohexyl functional groups, resulting in the release of eight hydrogen atoms, as shown in reaction 11. The ability to transfer eight hydrogen atoms gives the acetophenone/1-cyclohexylethanol couple a high hydrogen storage capacity of 6.3 wt %. Fusek et al. reported 1-cyclohexylethanol ECD to be sensitive to the Pt catalyst structure, and the reaction undergoes deactivation due to catalyst poisoning.^39^ They also identified acetophenone as one of the main products of 1-cyclohexylethanol ECD on Pt using spectroscopic techniques.^39^ These promising results open new doors to explore the 1-cyclohexylethanol ECD reactions in both fundamental and practical aspects for LOHC cycling applications. More importantly, ECH of acetophenone has to be developed to ensure the viability of LOHC cycling.

**Figure 5:**
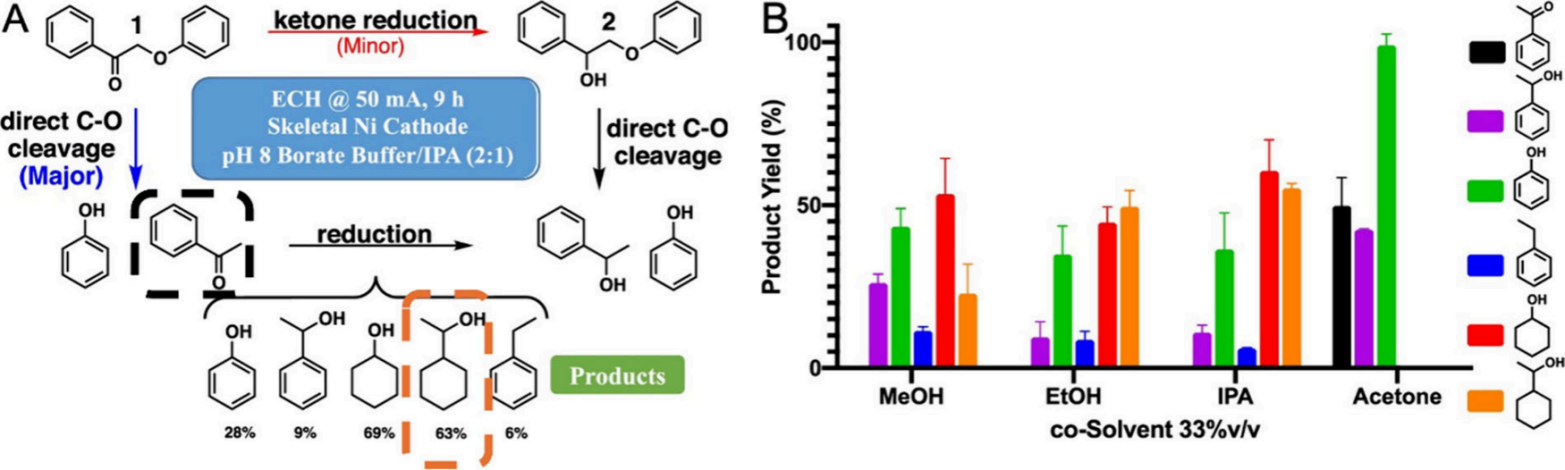
(A) Reaction mechanism pathway of 2-phenoxyacetophenone ECH on Ni, illustrating the possible pathway of acetophenone (black dashed box) as an intermediate product to form 1-cyclohexylethanol (orange dashed box) as one of its products. (B) Effect of the cosolvent on the product distribution during 2-phenoxyacetophenone ECH on Ni.^115^ Reaction conditions: applied current = 50 mA, borate buffer (pH 8)/IPA (2:1); reaction time = 9 h.^115^ Adapted with permission from ref (115). Copyright 2020 American Chemical Society)

Acetophenone ECH has been studied where 1-phenylethanol is reported as the main product on Pb^113^ and Pd/Pt.^114^ 1-Cyclohexylethanol has not yet been observed in the studies of acetophenone ECH. However, ECH studies on 2-phenoxyacetophenone, an ethyl dimer found on lignin, have been reported on Ni and in a borate buffer solution (pH 8).^115^ 1-Cyclohexylethanol was obtained with 63% product yield.^115^ The mechanism pathway involves cleavage of the dimer to form acetophenone, which is then further reduced to obtain 1-cyclohexylethanol as one of the end products, as shown in Figure 5A. On the contrary, 1-cyclohexylethanol was not obtained when reticulated vitreous carbon was used as the catalyst, indicating the role of catalyst material in acetophenone ECH.^115^ Finally, the cosolvent affected the product distribution, where 1-cyclohexylethanol was favored by ethanol and IPA cosolvents (Figure 5B).^115^ These findings show that acetophenone ECH could be tuned to selectively produce 1-cyclohexylethanol for LOHC cycling applications. Depending on the molecule and reaction conditions, hydrogen storage can be in an aromatic ring, in ketones/aldehydes, or in both aromatic rings and ketones/aldehydes. Thus, LOHCs can undergo electrochemical redox reactions involving hydrogen transfer at more than one functional group, resulting with a relatively high hydrogen loading capacity. Identifying and developing the electrochemical redox reactions of such molecules are needed to guide the development of electrochemical cycling for LOHCs.

Overall, electrochemical LOHC cycling provides a promising green strategy in the hydrogen cycling economy. Renewable electricity can be used to selectively enhance electrochemical reactions and product selectivity. The ability to generate in situ protons in ECH reactions eliminates the process units for molecular hydrogen production and handling. However, based on the current state of literature, further systematic studies are required for the development of efficient LOHC couples. The majority of the works reported are carried out at lower organic concentrations depending on the intent of the study. For an electrochemical LOHC cycling system to be utilized at a commercial scale, higher LOHC concentrations will be required to avoid negatively affecting the hydrogen storage capacity, particularly in transport applications. Challenges facing electrochemical reactions at higher concentrations include poor conductivity, higher organic membrane crossover, and catalyst deactivation from polymerization side reactions. There is a gap to understand and improve ECH and ECD reactions at higher concentrations by designing suitable catalysts, membranes, reactors, and electrolytes for prospective scaling of the electrochemical LOHC cycling technology. The development of electrocatalytic systems such as Pd membrane reactors will improve reaction conversions and product selectivity, ensuring efficient cycling of LOHCs. Integrating the catalyst with kinetic and mechanistic studies can optimize the reaction conditions, enhancing product selectivity and FE and lowering overpotentials. This approach will improve the reaction efficiency by facilitating selective conversion of reactants to the desired products while minimizing parasitic side reactions such as C–C, C–O, and C–N bond cleavages, polymerization, and HER and OER.

## Carbon Footprint and Energy Efficiency Analyses

The viability assessment of the LOHC cycling process should also be extended to provide good environmental practices and sustainability measures. The electrochemical LOHC cycling process offers an opportunity to reduce the carbon footprint by utilizing renewable electricity and avoiding the use of fossil fuels as an energy source for chemical reactions. Nevertheless, the whole chemical process must be evaluated for each reaction to determine whether there are net reductions in carbon emissions. Apart from carbon emissions, energy efficiency should be considered when developing electrochemical systems for LOHC cycling to ensure proper utilization of energy resources. In this section, a comparative carbon footprint analysis for the LOHC cycling methods is provided, assuming the same LOHC applications for both methods. In addition, an overview of energy efficiency in electrochemical reactions is provided to assess the practical use of electrochemical cycling applications for LOHC molecules.

### Carbon Footprint Analysis

Mitigating carbon emissions and ensuring clean energy accessibility are the primary driving goals for the development of sustainable energy technologies. A number of studies have been reported for the development of thermochemical reaction processes for LOHC cycling applications.^11,14−27,29^ Nevertheless, carbon emissions are still a major concern of these processes due to heating by fossil fuels. One of the major motivating factors for introducing electrochemical reactions for LOHC cycling is reducing carbon emissions by integrating the process with renewable electricity sources. Thus, for electrochemical LOHC cycling processes to be considered practically, they have to have a significantly lower carbon footprint than that of the thermochemical processes. In this section, we will perform a comparative analysis of the carbon footprint for the thermochemical, electrochemical, and hybrid LOHC cycling processes. Process simulations were run using *Aspen Plus* version 12.1 and acetone/IPA as the LOHC couple. The acetone/IPA couple was used as the LOHC couple model due to its extensively studied reactions in multiple applications. Also, acetone and IPA have a sufficient thermodynamic database in *Aspen Plus* and therefore can be used as a simple model approach. The property methods used in *Aspen* software were the Soave–Redlich–Kwong and nonrandom two-liquid models for the thermochemical reactors and distillation simulations, respectively. Assumptions made include the following: (i) Molecular H_2_ for the thermochemical and hybrid processes was obtained from water electrolysis, while the electrochemical process generated protons in situ. (ii) A total of 1 kmol/h of LOHC molecule was fed into the reactor. (iii) Natural gas was the fuel source for heating the streams, reactors, and distillation reboilers. (iv) Nonrenewable electricity is sourced from natural gas. (v) The carbon footprint for the production of starting materials (either acetone or IPA) was not considered because it is the same for all processes. (vi) The carbon footprint of water as a solvent or reactant was ignored. (vii) No joule heating of the electrochemical reactor occurred from ohmic losses. (viii) Electrochemical reactions run at thermodynamic redox potentials. (ix) Pumps and compressors are operated by electricity. (x) The final use of H_2_ is the same for both cycling methods. Details beyond those presented in this Perspective for the carbon footprint calculations can be found in ref (116).

#### Thermochemical LOHC Process

**Figure 6:**
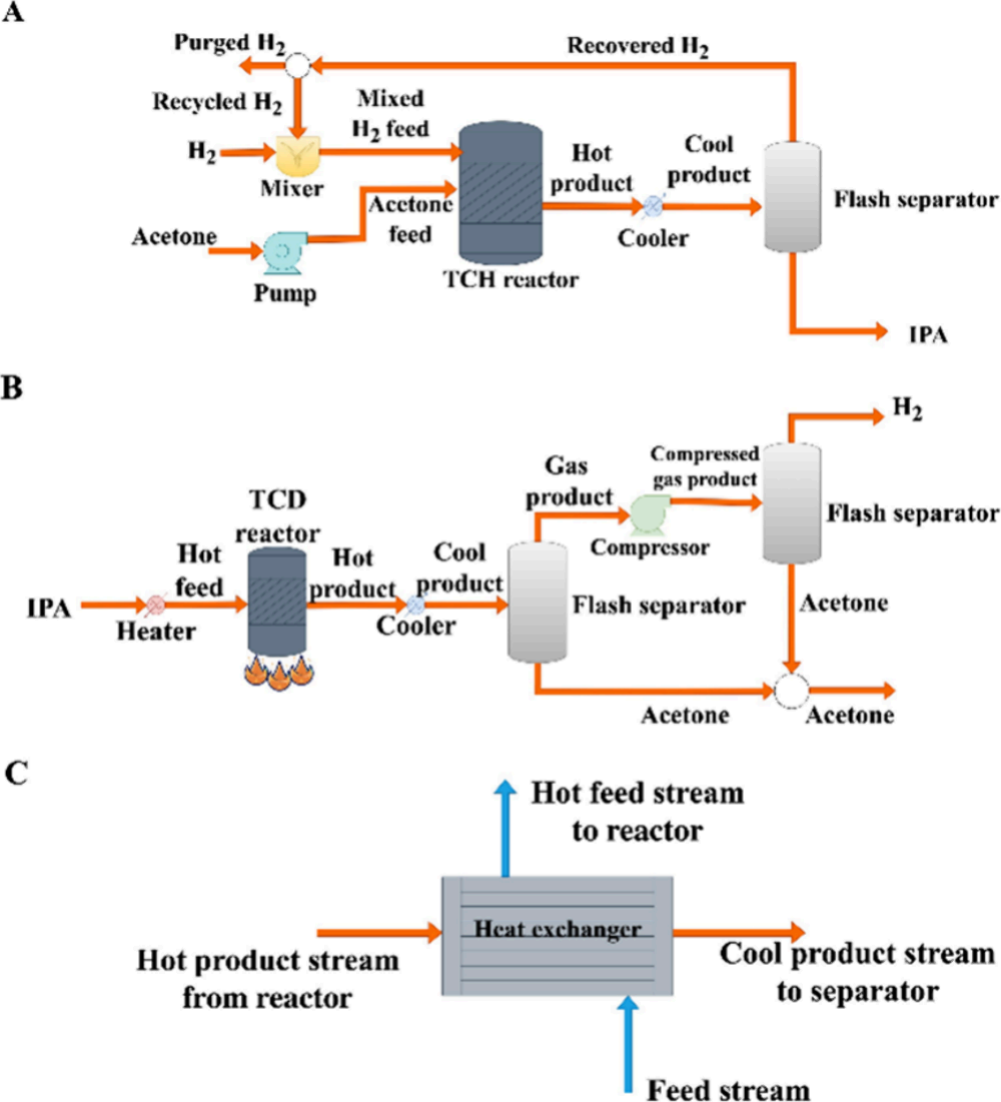
Process flow diagrams for (A) acetone TCH, (B) IPA TCD, and (C) heat recovery processes.

IPA can be commercially obtained from the TCH of acetone.^117^ Acetone TCH can be utilized as a method to store hydrogen in the LOHC cycling process. Acetone TCH can be done under continuous flow or batch reactor mode of operation.^118,119^ Various metal catalysts such as copper-, nickel-, and zinc-based can be utilized for acetone TCH.^118,119^ These catalysts can be in metallic alloy form with a support such as silica or alumina.^118,119^ Herein, acetone TCH was simulated at reaction conditions in the range of those reported from patent literature, obtaining at least 98% for both conversion and yield.^118,119^Figure 6A details the process flow for the acetone TCH simulation in *Aspen Plus*. The acetone feed containing 2 wt % water was pumped to the reaction pressure before being fed to the reactor. The presence of water in the acetone feed has been reported to reduce the formation of 4-methyl-2-pentanol and 2-methylpentane-2,4-diol occurring as side products.^118^ However, the water content should be low, preferably below 4 wt %, for easier product purification.^118^ H_2_ feed was also introduced to the reactor, resulting in a feed ratio of 1:4 acetone/H_2_ at the reactor inlet. For LOHC cycling applications, the ideal situation is to obtain 100% conversion and selectivity to ensure a continuous run of cycles in storing and releasing hydrogen in LOHCs. As a hypothetical comparison, the reactor was modeled as an adiabatic RStoic in *Aspen Plus*, with the reaction taking place at 98% conversion of acetone and 100% IPA selectivity. The adiabatic reactor had an outlet temperature of 120 °C, maintaining a pressure of 2 MPa. The obtained products from the reactor outlet were then cooled to 25 °C before entering the flash separator, where the gaseous H_2_ was separated from the liquid components in the product mixture. The recovered H_2_ had a purity of 90 wt % with the rest comprised of unreacted acetone, water, and IPA product. A total of 7% of the recovered H_2_ was purged out of the system to avoid accumulation of water and IPA in the feed stream. The remaining amount of recovered H_2_ was recycled back to the H_2_ feed stream to react with acetone and also serve as a coolant for the exothermic TCH reaction. The results obtained from the simulation show that 500 kJ/h was required for pump work. The acetone TCH simulation process resulted in a production of 58.91 kg/h of IPA with 97 wt %, equivalent to 0.83 kg/h of H_2_ stored. From the simulation results, calculations were performed to determine the carbon footprint of the overall TCH process. The CO_2_ emission coefficient of natural gas combustion fuel was obtained from the Energy Information Administration and reported to be 5.02 × 10^–5^ kg_CO_2__/kJ.^120^ The carbon footprint of H_2_ production from water electrolysis using wind-sourced renewable electricity is 0.7 kg_CO_2__/kg_H_2__, which is an estimated average from literature reports.^121−123^ The carbon footprint of H_2_ production from water electrolysis was obtained assuming that 50 kWh is consumed per kg of H_2_ in a typical water electrolyzer operating at 80% efficiency.^124^ Using nonrenewable electricity obtained from natural gas, the carbon footprint for H_2_ production by water electrolysis is 22 kg_CO_2__/kg_H_2__. This carbon footprint value is also similar to that reported by Parkinson et al.^125^ Consequently, the acetone TCH process had a carbon footprint of 2.2 kg_CO_2__/kg_H_2__ stored when electricity was from a renewable source, as shown in Table 2. The upstream carbon footprint of H_2_ largely contributed to the TCH carbon footprint. Further details and calculations for the carbon footprint of TCH with different sources of energy are provided in Table 2 and a preprint.^116^ The carbon footprint significantly increased when hydrogen was sourced from nonrenewable sources such as steam methane reforming or fossil fuel electricity. The carbon footprint from acetone TCH when hydrogen was produced from water electrolysis with nonrenewable electricity was 69.4 kg_CO_2__/kg_H_2__ stored, which is 30 times greater than that when renewable electricity was utilized in water electrolysis for hydrogen production.

**Table 2:** Comparative Carbon Footprint for LOHC Cycling Processes

H_2_storage/release process	scenarios	carbon footprint with renewable electricity (kg_CO_2__/kg_H_2__ stored or released)	carbon footprint with nonrenewable electricity (kg_CO_2__/kg_H_2__ stored or released)	carbon footprint with renewable electricity (kg_CO_2__/kg_LOHC_ produced)	carbon footprint with nonrenewable electricity (kg_CO_2__/kg_LOHC_ produced)
acetone TCH		2.2	69.4	0.03	0.98
IPA TCD	with heat recovery	3.0	4.9	0.10	0.16
	without heat recovery	4.3	6.3	0.14	0.21
acetone ECH	2 M acetone (water)	23.7	51.2	0.34	0.71
	pure acetone or diluted acetone^b^ without separation process	0.80	28.3	0.01	0.39
IPA ECD^a^	2 M IPA (1 M KOH)	29.2	29.2	1.01	1.01
	2 M IPA (water)	5.8	5.8	0.20	0.20
	2 M IPA without a separation process (1 M KOH)	23.4	23.4	23.4	23.4
	pure IPA or diluted IPA^a^ in water without a separation process	0.00	0.00	0.00	0.00

a The IPA ECD carbon footprint for these models is independent of the electricity source.

b Diluted IPA/acetone represents the range of IPA/acetone concentrations in aqueous (water only) electrolyte.

Dehydrogenation reactions, being endothermic, often require higher operating temperatures than hydrogenation reactions, resulting in higher energy requirements. IPA TCD used to be the main process for the industrial production of acetone and was later replaced by cumene oxidation as the main acetone production process,^126^ which has cheaper raw materials and also coproduces phenol as a valuable product. However, IPA TCD is still currently deployed to a minor extent to produce acetone with high purity.^126,127^ IPA TCD can be utilized for hydrogen release during the LOHC cycling process. IPA TCD has been reported to occur at temperatures in the range of 200–450 °C and at atmospheric pressure using a fixed-bed reactor.^126,128^ In this work, IPA TCD was simulated using *Aspen Plus*, and the process flow is shown in Figure 6B. The reaction conditions used were adopted from patent literature where 90% conversion and 99% selectivity were observed on a Cu/Zn/Cr/Al_2_O_3_ trimetallic catalyst.^128^ In this simulation, the reactor was operated at 420 °C and 0.1 MPa. The feed was preheated to the reaction temperature before entering the reactor. The reactor was modeled as an isothermal RStoic, producing a 90% conversion and 100% selectivity. The simulation results showed that IPA TCD requires more energy than acetone TCH due to high operating temperatures and the reaction being endothermic. The energy required to heat the feed to the reaction temperature was 96900 kJ/h. Moreover, 53900 kJ/h was required for the reactor to maintain the reaction temperature. A heat exchanger model was applied to analyze how much of the heat could be recovered from the hot product stream, as shown in Figure 6C. From the heat recovery simulation, 60% of the energy could be recovered from the hot product stream with a pinch point temperature of 15 °C. With the utilization of a heat exchanger, 49900 kJ/h was the net energy required to heat the feed stream. The reactor outlet products were cooled to 25 °C before entering a series of flash separators. The first flash separator was at 25 °C and 0.1 MPa, where a mixture of hydrogen and acetone were obtained in the vapor top product. The hydrogen and acetone gas mixture was compressed to 8.5 MPa and entered the second flash separator operating at a low temperature of 5 °C and high pressure of 8 MPa. H_2_ was obtained as the top product with a purity of 95 wt %, while acetone with composition of 90 wt % was obtained from the bottom products. The carbon footprint from the overall TCD process when renewable electricity and heat recovery was utilized was 0.1 kg of CO_2_ per 1 kg of acetone produced, equivalent to 3 kg of CO_2_ released per 1 kg of H_2_ generated. Further information on various IPA TCD scenarios for comparison is provided in Table 2, and respective calculations are described in the preprint.^116^ In general, the carbon footprint for storing and releasing hydrogen via thermochemical LOHC cycling process simulation with renewable and nonrenewable electricity was obtained to be 5.2 and 74.3 kg_CO_2__/kg_H_2__, respectively. A large part of the carbon footprint is attributed by the endothermic TCD reaction and the H_2_ upstream carbon footprint for the TCH reaction.

#### Electrochemical Process

**Figure 7:**
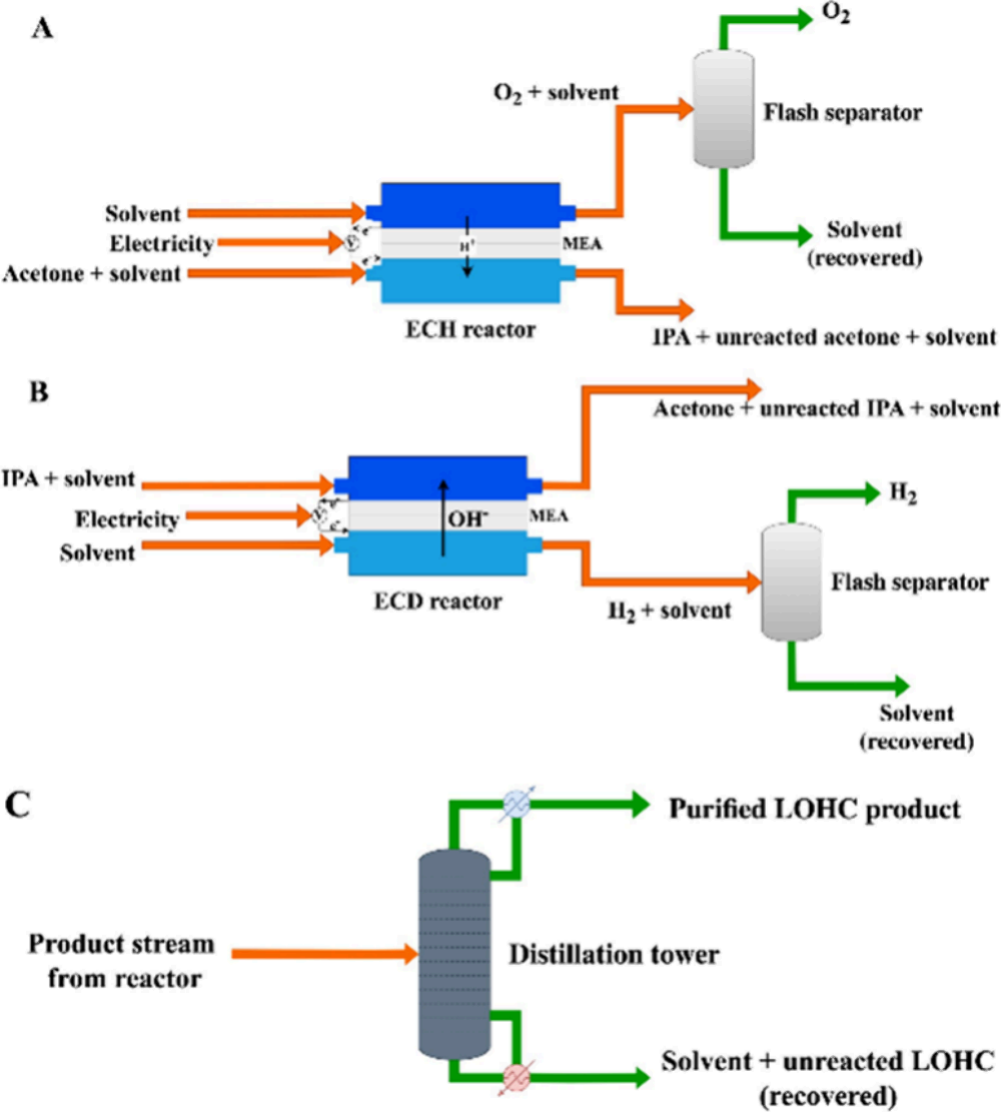
Process flow diagrams for (A) acetone ECH, (B) IPA ECD, and (C) the distillation separation unit for LOHC product purification.

In this section, the electrochemical cycling processes of acetone/IPA were simulated to determine the carbon footprint in comparison with the thermochemical processes. Acetone ECH has been performed in an electrochemical flow reactor with reported IPA selectivity of up to 100% and acetone concentrations of up to 7 M on Pt/Ru in the literature.^64^ The acetone ECH simulation was performed on the basis of 2 M acetone concentration in water due to the better results observed at lower acetone concentrations in the acetone ECH system.^64^Figure 7A illustrates the details of the acetone ECH process using a process flow diagram. The ECH reactor was modeled as a block with 98% conversion and 100% IPA selectivity to match that of the TCH process. The reaction was an overall process in which acetone ECH operated at the cathode coupled with anode OER (reactions 12–14). Thus, the applied cell voltage was assumed to be 1.11 V, which is the theoretical thermodynamic potential of acetone ECH. However, in a practical system, the applied voltage will be higher considering the voltage losses and catalyst used for the reaction.
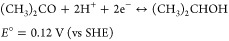
12
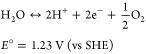
13
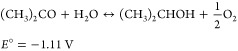
14

The reactions were modeled at 25 °C and 0.1 MPa, removing the need of heating the reactor or the feed stream. Assuming the application of renewable electricity to drive the reaction, the acetone ECH carbon footprint was calculated as 0.8 kg_CO_2__/kg_H_2__ stored. However, this value is incomplete considering that there may be a need to purify the LOHC product for other uses or in a case where ECD will be required to be performed in a different electrolyte. Transportation or storage may also be more economical at higher concentrations. Significant quantities of energy will be required for a distillation process to separate the product from the electrolyte/solvent. A distillation tower was modeled as a RadFrac in *Aspen* to separate the product stream components, as shown in Figure 7C. IPA was obtained in the distillate stream, where it forms an azeotropic mixture with water at 79 °C having a purity of 85 wt %. The amount of energy required for the separation process for 2 M acetone ECH was 372000 kJ/h. Further purification can be performed using a liquid entrant such as ethylene glycol or dimethyl sulfoxide to obtain IPA purity as high as 99 wt %.^129^ However, the use of such solvents will come at the cost of carbon emissions considering their upstream processes and so were not utilized. The overall carbon footprint for 2 M acetone ECH with distillation was 23.7 kg_CO_2__/kg_H_2__ stored (0.34 kg of CO_2_ per 1 kg of IPA produced), which is about 30 times more than ECH without separation, as shown in Table 2. Furthermore, the 2 M acetone ECH carbon footprint was 10 times greater than that of the corresponding TCH process when separations were included. Product separation from the electrolyte solution is the main factor attributed to larger CO_2_ emissions in acetone ECH.

**Figure 8:**
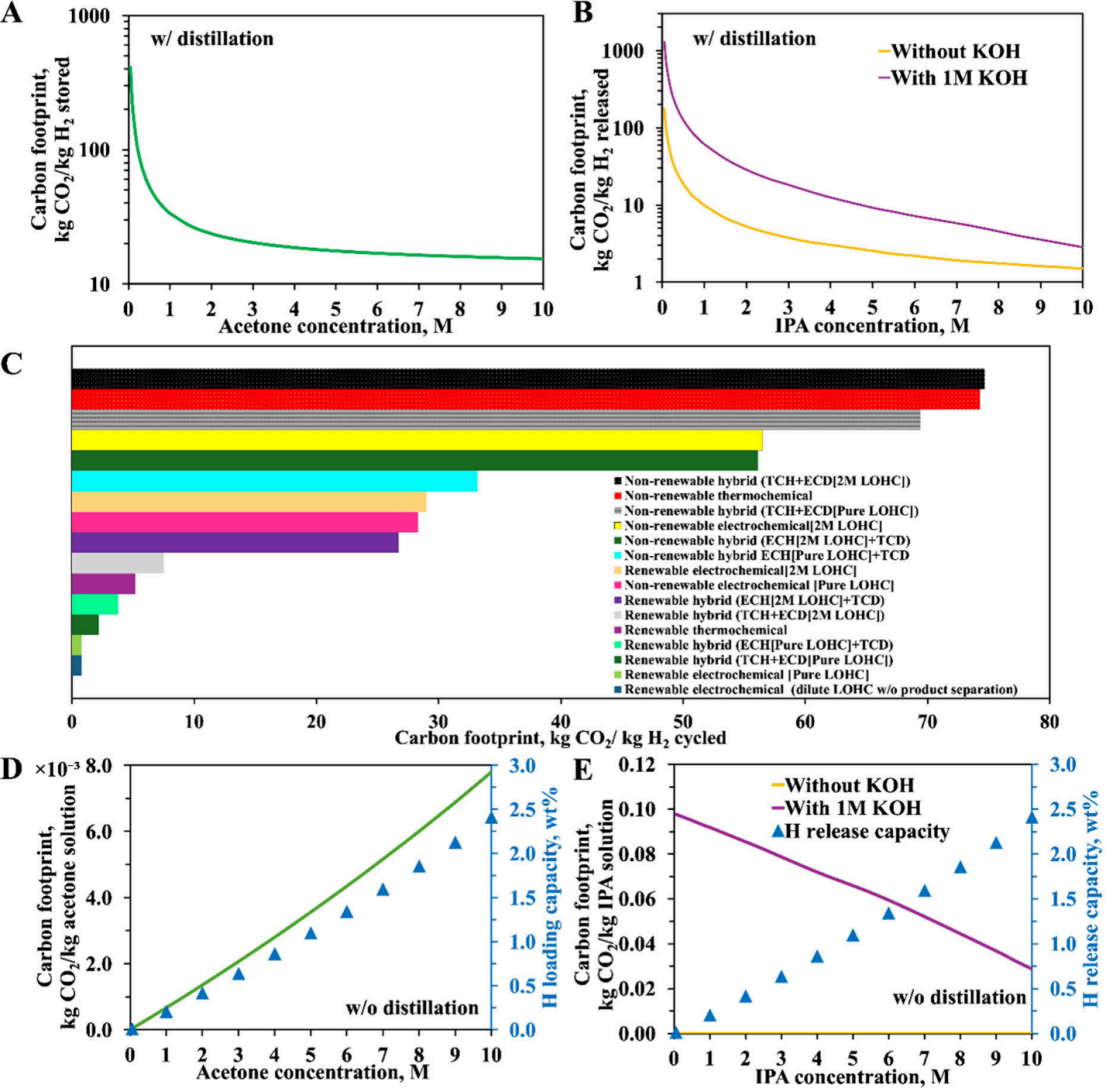
(A) Comparative sensitivity analysis of the carbon footprint per H_2_ stored as a function of the acetone concentration in water during the ECH process. (B) Comparative sensitivity analysis of the carbon footprint per H_2_ released. The carbon footprint analysis is shown for two cases (with and without 1 M KOH during the ECD process). (C) Comparison of the carbon footprint of LOHC cycling systems under various scenarios and electricity sources. [2 M LOHC] indicates that the electrochemical process was simulated with 2 M LOHC concentration in water. (D) Comparative sensitivity analysis of the carbon footprint per amount of acetone solution (green line) and hydrogen loading capacity (blue triangles) as a function of the acetone concentration in water. (E) Comparative sensitivity analysis of the carbon footprint per amount of IPA solution (purple and orange lines) and hydrogen release capacity (blue triangles) as a function of the IPA concentration in water.

A sensitivity analysis was also performed to investigate the effect of the acetone concentration in ECH on the carbon footprint, including separation. The analysis was performed at a constant acetone feed rate of 1 kmol/h and 84.8 wt % IPA in the product stream. In Figure 8A, the carbon footprint decreases as the concentration of acetone increases from 0.05 to 10 M. The carbon footprint of 0.05 M acetone was 410 kg of CO_2_ released per 1 kg of H_2_ stored, while at 10 M acetone, 15.4 kg of CO_2_ was released per 1 kg of H_2_ stored. The decrease of CO_2_ is due to less water solvent used at higher concentrations and therefore requires less energy for product separation. It should also be noted that increasing the acetone concentration affects the reaction performance. Green et al. performed acetone ECH in a range of approximately 0.5–7 M acetone and observed a slight decrease in conversion and current efficiency with increasing concentration at the concentration range studied.^64^ Further studies are needed for the development of ECH reactions that operate at high concentrations for LOHC applications to make them more viable.

Additionally, the electrolyte/solvent lowers the hydrogen storage capacity of the LOHC couple, limiting their transportation and mobility application viability. For instance, the hydrogen storage capacity of the acetone/IPA couple in its pure form is 3.3 wt %. However, diluting the LOHC in electrolyte lowers the hydrogen storage capacity, as shown in Figure 8D,E. Decreasing the acetone or IPA concentration from 10 to 0.05 M lowered the hydrogen loading/release capacity from 2.4 to 0.01 wt %.

IPA ECD was also simulated, and the process is illustrated in Figure 7B. IPA ECD has been demonstrated for DAFC coupled with ORR at the cathode.^41,68,73,130^ IPA in water can be used as the anolyte feed.^74^ IPA ECD has also been performed under alkaline conditions (1 M KOH) in a two-compartment electrochemical cell coupled with HER on the cathode.^72^ Herein, IPA ECD was coupled with HER where IPA consumes hydroxyl ions to dehydrogenate and form acetone (reaction 15). On the counter side, hydrogen is obtained on the cathode by HER (reaction 16) and releasing hydroxyl ions that transfer to the anode via an anion-exchange membrane. In this simulation, the carbon footprint of IPA ECD was demonstrated with and without 1 M KOH in the electrolyte solution. IPA was fed to the reactor and did not require heating because the reaction was assumed to operate at 25 °C and 0.1 MPa. The overall IPA ECD given in reaction 17 shows that the reaction occurs spontaneously and electricity will not be required to run the reaction. It should be acknowledged that the practical operating conditions including overpotentials may require applied voltage to run the reaction (not modeled here).
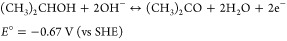
15
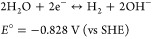
16
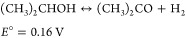
17

The reaction conversion (90%) and selectivity (100%) were the same as those of IPA TCD for a direct comparison. The product stream was first introduced into a flash to separate the obtained hydrogen gas from the liquid electrolyte. The flash was modeled to obtain H_2_ with a purity of 95 wt %, same as that of the IPA TCD process. A separation process was also modeled as in Figure 7C using a RadFrac from *Aspen Plus*. The anolyte product stream composed of acetone, unreacted IPA, and electrolyte solvent entered the distillation tower for purification, where acetone with 90 wt % was obtained as the top product, while an electrolyte solution was recovered from the bottom stream of the tower. Simulation was performed under two different scenarios using water as the only electrolyte and using 1 M KOH electrolyte to demonstrate the effect of KOH on the carbon footprint. For the simulations using a 1 M KOH alkaline solution, the carbon footprint of KOH was also considered where 1.8 kg of CO_2_ was released for a 1 kg of KOH.^131,132^ In the case where product separation was not required, 2 M IPA ECD had a zero carbon footprint when only water was used as the electrolyte and 23.4 kg_CO_2__/kg_H_2__ was released when 1 M aqueous KOH was used as the electrolyte (Table 2). When product separation was needed, the ECD of 2 M IPA in water had a carbon footprint of 5.8 kg_CO_2__/kg_H_2__ released. When ECD was performed using a 1 M aqueous KOH electrolyte, the carbon footprint increased 5 times to 29.2 kg_CO_2__/kg_H_2__ released, as shown in Table 2. The carbon footprint of the upstream processes for producing KOH or other solvents will largely determine the overall carbon emissions for electrochemical LOHC cycling, and methods requiring green solvents/electrolytes will need to be investigated and developed. The carbon footprint of 2 M IPA ECD under neutral conditions is almost twice that of the IPA TCD process. However, at concentrations greater than 6 M IPA, the ECD carbon footprint becomes lower than that of the TCD process. More details and comparisons of the carbon footprint with different scenarios are provided in Table 2 and the preprint.^116^ A sensitivity analysis was also performed for IPA ECD, and the same trend of acetone ECH was observed, as seen in Figure 8B. The carbon footprint decreased an order of magnitude with an increase of the IPA concentration from 0.05 to 10 M. With water as the only electrolyte, 0.05 M IPA exhibited a carbon footprint of 178 kg_CO_2__/kg_H_2__ released, which decreased to 2 kg_CO_2__/kg_H_2__ released at 10 M IPA concentration. In a 1 M KOH electrolyte, the carbon footprint also significantly decreased from 1290 to 3 kg_CO_2__/kg_H_2__ released when the IPA concentration was increased from 0.05 to 10 M. IPA and alcohols in general have shown promising performances on ECD reactions in alkaline conditions and offer room for use of a wide range of catalysts. However, the trade-offs with the high carbon footprint comes with on overall IPA ECD and should also be considered when developing ECD processes for LOHC cycling. Further developments and studies on using high substrate concentrations in ECD will also help in the development of ECD in the LOHC cycling for energy transport and mobility applications.

For stationary storage applications, acetone/IPA systems offer the advantage of a smaller carbon footprint (Figure 8C and Table 2). Because stationary storage is not primarily limited by tank capacity, separation of the dilute acetone or IPA solution is not necessarily required, eliminating the need for a downstream separation unit. The carbon footprint of the dilute acetone/IPA couple in water electrolyte and without distillation was 0.81 kg_CO_2__/kg_H_2__ cycled (Figure 8C). Carbon footprint estimates assumed a constant LOHC feed with varying concentrations, meaning that the solution volume changes inversely with the concentration. While Figure 8D shows that the carbon footprint per unit of acetone solution rose with increased acetone concentration when no downstream distillation was applied, the footprint is in grams of CO_2_ per kilogram of solution cycled. The more concentrated solutions are more favorable operationally with a minimal footprint impact. The carbon footprint of IPA ECD in water alone was zero because no emissions resulted from the reaction itself (Figure 8E). However, with 1 M KOH, the carbon footprint increased to 0.098 kgCO_2_/kgIPA solution at 0.05M, owing to KOH carbon footprint (Figure 8E). Higher IPA concentrations (lower amount of IPA solution) decreased the footprint to 0.029 kg of CO_2_ per 1 kg of IPA solution at 10 M as less KOH was used (Figure 8E). Overall, LOHC cycling for stationary storage without separation yields a lower carbon footprint. A higher amount of solution favors greater hydrogen storage capacity and less carbon footprint per unit. The trade-offs between the tank capacity, LOHCs concentration, and electrolyte used should highly be considered when developing electrochemical cycling systems for stationary storage applications.

#### Hybrid Process

A hybrid process for LOHC has been discussed as an alternative to LOHC cycling. For a hybrid LOHC cycling process, the separation unit for the electrochemical process would be needed to provide a high-purity LOHC reactant in a thermochemical process. Separation processes could lead to a substantial carbon footprint associated with a hybrid LOHC cycling process. Electrochemical reactions operated with pure LOHC molecules will allow for strategic integration with thermochemical reactions for LOHC cycling. Figure 8C shows a carbon footprint comparison of different LOHC cycling processes under different scenarios. When using renewable energy sources, the carbon footprint of hybrid LOHC cycling, where TCH and ECD of pure LOHC are the hydrogen loading and releasing systems, respectively, is 2.2 kg_CO_2__/kg_H_2__ cycled. On the other hand, when hydrogen loading is via a pure acetone ECH process while the hydrogen release is via IPA TCD (60% heat recovered), the carbon footprint obtained is 3.8 kg_CO_2__/kg_H_2__ cycled. TCD reaction requires higher energy to operate at high temperatures and can therefore be replaced by ECD reaction only when concentrations greater than 4 and 9 M are used for water only and 1 M KOH electrolyte, respectively. The preprint^116^ details further information regarding the carbon footprint of different LOHC cycling scenarios. The TCH and ECD hybrid LOHC cycling could offer a lower carbon emission system compared to the thermochemical LOHC cycling but only when pure LOHC is used in the reactions. From the simulation results, a hybrid process would be feasible only when the electrochemical processes are conducted at higher concentrations. The product from the electrochemical reaction has to be separated from the electrolyte solution in order to be utilized in the thermochemical process.

### Energy Efficiency

Energy efficiency (EE) is another major criterion that can help with the implementation of electrochemical cycling of LOHC technology at the industrial level. In general, the voltage applied to the cell drives electrochemical reactions. The cell potential accounts for standard redox potentials, ohmic losses, cathodic and anodic overpotentials, and mass transfer limitations (eq I). Operating potentials are usually higher than theoretical potentials due to operational losses, which include voltage losses from ohmic resistance and parasitic side reactions. Not all energy is used for the desired reactions.^64^ Higher currents can be achieved at higher potentials. However, high currents can be due to parasitic side reactions, not only the desired reaction, and FE accounts for the current utilized toward the desired reaction. In addition, the energy efficiency of the specific LOHC systems can be assessed in terms of voltage at a given current. The EE of LOHC can be obtained using the ratio of the theoretical equilibrium potential of the cell to the operating cell potential at a given current density to drive ECH or ECD reactions and FE (eq II). In general, energy efficiency is a quotient of the theoretically required power to carry out the reaction and the actual power used.^133^
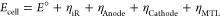
I
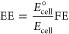
IIwhere *E*_cell_ is the cell potential, *E*° is the standard cell potential, η_iR_ is ohmic losses caused by electrolyte and contact resistances, η_Anode_ and η_Cathode_ are losses due to the anodic and cathodic reactions, respectively, and η_MTL_ is loss due to mass-transfer limitations.

EE describes how well the system performs compared to the theoretical potential values.^64^ For instance, if the electrochemical reactions are slow, then the system would require higher overpotentials to drive the desired reactions. In addition, electrode degradation can cause increased resistance at the electrode–electrolyte interface, which will require higher overpotentials.^134^ Improving the kinetics of the desired reactions can provide a solution to maintain the enhanced EE. Also, tailoring the electrode structure and composition can enhance the reaction kinetics. For example, using catalysts with high surface area and activity can facilitate faster reaction rates and improve the mass transport of reactants to the electrode surface.

As an example, consider the acetone/IPA couple, where the standard cell potential for the acetone/IPA reduction ECH reaction is −1.11 V. However, the operating cell potential for the acetone/IPA couple ECH reaction has been reported to be −1.3 to −1.6 V.^64,72^ Thus, if a reactor operates at a FE of 88%,^135^ it would yield an EE of 74% at −1.3 V (eq III), which means only 74% of the energy used goes into hydrogenation of acetone to IPA, and the rest of the energy is used for unwanted reactions or heat generation.
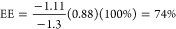
III

However, operating at −1.3 V would yield small current densities (5 mA/cm^2^),^55^ and higher current densities can be achieved by increasing the operating potential. For instance, operating at −1.6 V has yielded 45 mA/cm^2^,^55^ which would reduce EE to 60.5%. It is noteworthy that even though applying higher voltage can increase the current density to 45 mA/cm^2^, it is still far from industrial level current densities (>100 mA/cm^2^).^133^ Thus, EE is subject to a trade-off between higher reaction rates and economic aspects of the technology. Further reaction improvements can also positively impact the EE. It is important to note that the EE discussed here focuses solely on the electrochemical cycling reactions. The energy efficiencies of other processes, such as shipping and product purification required to complete the full electrochemical cycle, were not considered. Wang et al. evaluated the EE evaluation of thermocycling in an LOHC system using predicted properties of an ethylcarbazole derivative, including shipping considerations.^136^ Based on their assumptions and calculations (excluding shipping), the EE of ethylcarbazole-based LOHC thermochemical reactions were 72% without heat recovery and 92% with heat recovery, assuming 88% completion of LOHC dehydrogenation. The dehydrogenation rate significantly influences the EE of thermochemical cycling reactions, with lower rates leading to decreased efficiency.^25,136,137^ One should also be cautious about comparing EE across the technological maturity level, with most electrochemical reactions being far less optimized than their corresponding thermochemical reactions to date.

In addition to EE, specific electricity consumption (SEC; eq IV) can be used to compare LOHC systems operating under different conditions. SEC utilizes operating power (*W*), which is the product of the cell potential and current (IU_cell_), and production rate (PR_i_), which can be either a molar or gravimetric rate. The ratio of the power to production rate would yield the amount of electrical energy utilized by the system to produce a certain amount of the desired product.^138^
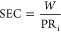
IV

SEC can be used to compare two distinct LOHC couples operating at various conditions based on the overall energy efficiency of the reactions. In general, SEC indicates that higher currents negatively affect the energy efficiency of the electrochemical reactions. This is because higher currents are not always indicative of high rates of desired reactions; instead, they can partially result from parasitic side reactions, such as HER in the case of hydrogenation reactions. Therefore, high FE values ensure that the energy consumed, for example, in ECH, is used toward producing the desired products and not driving side reactions like HER. Overall, designing effective electrocatalytic systems is crucial to achieving optimal EE and SEC values, which is one of the ultimate benchmarks of how likely the electrochemical cycling LOHC technology can be accepted and implemented at industrial levels.

## Toxicity and Water Usage

### Toxicity

The environmental and health impacts of LOHC molecules are crucial factors for practical hydrogen storage feasibility. Electrochemically active LOHC couples such as formaldehyde/methanol, acetonitrile/ethylamine, and benzene/cyclohexane have demonstrated a good hydrogen storage capacity. However, molecules such as formaldehyde and benzene are considered highly toxic, creating concerns for their utilization as LOHC candidates. For instance, formaldehyde is a known carcinogen^96^ with a high vapor pressure, increasing exposure risks. Similarly, benzene has been evaluated, and studies show its carcinogenic effects, leading to strict regulations for its usage.^51^ In addition, acetonitrile can produce toxic VOCs and has a considerable occupational exposure risk.^58^ There are limited studies on the toxicology profiles of many LOHC molecules. With the growing field of LOHCs for hydrogen storage and transportation, parallel toxicity studies are required to ensure sustainable applications of LOHC molecules. Markiewicz et al. performed a critical assessment for LOHC couples to reduce the risk assessment uncertainty and ensure that preliminary safety measures are acknowledged before advancing with a particular LOHC couple.^139^ They proposed numerical models to provide guidelines as an initial step for risk assessment and to later be justified by experimental studies. Studies to assess the environmental and health effects of LOHC applied for electrochemical cycling will be crucial to providing feasible routes for process development. Properties such as biodegradability, water solubility, volatility, and reactivity will be important to determining the environmental risks of LOHCs as well.

### Water Usage

Water is a precious material, and electrochemical reactions are often carried out in aqueous-based electrolytes. Regardless of the method for LOHC cycling, water is commonly used as a hydrogen source. However, using aqueous-based electrolytes increases water usage in the electrochemical approach of LOHC cycling. In addition, as the carbon footprint analysis showed above, using highly diluted LOHC systems leads to greater CO_2_ emissions related to separation processes. Therefore, minimizing water as a solvent by using highly concentrated LOHC solutions would enable utilization of the electrochemical LOHC process without a separation process. Also, operating at high concentrations would allow one to increase the amount of hydrogen storage per gravimetric unit of LOHC compound, thus decreasing additional cost related with the transport and storage of LOHCs.

## Recommendations and Conclusions

In this work, selected electrochemically active molecules have been reviewed as candidates for the electrochemical LOHC cycling process. A good LOHC couple for electrochemical cycling should display a good hydrogen storage capacity, high reaction rates, selectivity, and FE to avoid the formation of side products and enable multiple cycles for long-term hydrogen storage and transportation. Further research on enhancing electrochemical reaction kinetics and mechanisms is necessary to determine optimal reaction conditions. Also, the development of novel electrocatalytic systems is required to achieve high conversion, selectivity, and FE for large-scale operations of electrochemical LOHC cycling. The distillation separation unit is the primary energy-demanding part of the electrochemical cycling process when there is a need to purify the LOHC product. It is necessary that LOHC couples be able to undergo ECH/ECD at high concentrations to reduce the amount of solvent used in ECH/ECD processes. Reducing the solvent amount will lessen the amount of energy needed for purification or eliminate the need for separation altogether. It will also increase the hydrogen loading of the system. On the other hand, key factors for LOHC cycling such as catalyst stability and energy efficiency of the reaction can be negatively impacted at high LOHC concentrations. Alternatively, using the same electrolyte for both ECH and ECD in applications, such as stationary storage, eliminates the need for separation processes. The carbon footprint analysis done in this work showed that electrochemical cycling of the LOHC process can be more sustainable in terms of carbon emissions compared to the thermochemical and hybrid cycling of LOHC processes when distillation is not required. To achieve the lowest carbon footprint, the electrochemical LOHC cycling method needs to be coupled with renewable electricity and does not require a LOHC downstream separation process. Furthermore, a holistic view of the overall electrochemical LOHC cycling process should not be overlooked to ensure that safe and sustainable operations are developed. The use of environmentally benign LOHC molecules and less water in the electrochemical cycling process will pave a more sustainable path toward the hydrogen economy. Environmental risk assessment for the electrochemical cycling LOHC potential candidates should be conducted as a preliminary step to ensure future sustainable practices. Overall, electrochemical cycling of the LOHC process offers a sustainable pathway for hydrogen storage, and its viability will predominantly depend on the growth and development of renewable energy sources. Electrochemical LOHC cycling application would be crucial for managing variable renewable energy sources and providing a pathway for equal access of energy throughout the world.
